# Protective Effect of Lactic Acid Bacteria Isolated from Ripened Foods Against *Listeria monocytogenes* in Plant-Based Fermented Dry-Cured Sausages

**DOI:** 10.3390/foods14091491

**Published:** 2025-04-24

**Authors:** José M. Martín-Miguélez, Cristina Castaño, Josué Delgado, Lary Souza Olegario, Alberto González-Mohino

**Affiliations:** 1 Higiene y Seguridad Alimentaria, Instituto de Investigación de Carne y Productos Cárnicos (IProCar), Facultad de Veterinaria, Universidad de Extremadura, Avda. de las Ciencias s/n, 10003 Cáceres, Spain; jmmm@unex.es (J.M.M.-M.); cristinacs@unex.es (C.C.); 2 Tecnología de los Alimentos, Instituto de Investigación de Carne y Productos Cárnicos (IProCar), Facultad de Veterinaria, Universidad de Extremadura, Avda. de las Ciencias s/n, 10003 Cáceres, Spain; laryolegario@unex.es (L.S.O.); albertogj@unex.es (A.G.-M.)

**Keywords:** biocontrol, clean label, meat product analog, plant-based analog, ready to eat

## Abstract

The aim of the study was to use a commonly employed technology in the meat industry, the inoculation of a biocontrol starter, in the processing of a plant-based fermented dry-cured sausage analog to improve its safety against possible *Listeria monocytogenes* contamination. Challenge tests were used to select suitable lactic acid bacteria (LAB) for the analog under industrial production conditions. First, 20 LAB strains were tested in vitro and five of them were further tested by stuffing the ingredients under industrial conditions. The *L. monocytogenes* counts highlighted *Latilactobacillus sakei* 205 as the most protective one, achieving a reduction of 2.6 log CFU/g. Further, a triangular test and Check-All-That-Apply test were performed to understand the organoleptic differences that could be expected in the final product. The batch inoculated with *Llb. sakei* 205 did not show any sensory differences from the commercial batch. Therefore, *Llb. sakei* 205 was identified as a potential protective starter due to the microbiological and sensory results. This pioneering study applied biocontrol starters to plant-based meat analogs, aligning with clean-label trends.

## 1. Introduction

The major requirement when developing meat analogs is a technological development that can mimic the target product in texture, color, and flavor [1]. However, food safety is an underexplored challenge, since the potential risks of these novel products to the consumers are unknown. Meat analogs, with a pH near 7, are generally susceptible to microbial contamination, although pH modification can improve their technological performance [2,3]. Meat analogs are susceptible to the same microbiological contamination as the meat products they mimic [4]. This is especially a problem for Ready-To-Eat (RTE) products since these foods do not undergo heat treatment, which inactivates most biological hazards, like meat products do [5].

*Listeria monocytogenes* caused 39.3% of the deaths from foodborne diseases in 2023 according to the EFSA (European Food Safety Authority) data, with the main food vehicle being RTE meat products [6]. *L. monocytogenes* is a facultatively anaerobic, non-spore-forming, psychrotrophic, Gram-positive bacterium whose flagellar motility depends on the temperature of the medium it is in [7]. This pathogenic microorganism is ubiquitous, which allows it to reach food during processing or through the raw materials being used. It is able to develop at refrigeration temperatures, thus posing a hazard in refrigerated RTE foods [8]. Listeriosis outbreaks in RTE analogs have already taken place [9]. In addition to food safety problem, its presence is also a commercial handicap since certain countries such as the United States of America require the absence of this microorganism in 25 g of all imported foods [10].

The use of additives and preservatives in meat analogs is quite common, with the aim of achieving safer products and aromas, flavors, colors, and textures reminiscent of the meat products they are imitating [11,12]. On the other hand, there is a general rejection of foods containing additives that has been dragging on for years [13,14,15]. Additives or other sapid ingredients are commonly used to mask vegetable flavors in the formulation of analogs, but fermentation has also been described as a useful strategy that could help to reduce the amount of additives in meat analogs in order to adapt to clean label trends [16,17]. Moreover, using microorganisms as protective cultures is one of the cheapest and simplest strategies to minimize the proliferation of *L. monocytogenes* and other pathogenic microorganisms, as well as spoilers [18]. The use of lactic acid bacteria (LAB) as protective cultures is currently expanding due to their status as Qualified Presumption of Safety (QPS) [19,20]. The utilization of protective cultures in RTE meat products has been widely found to be able to control *L. monocytogenes* development in dry-cured fermented sausages due to different inhibition mechanisms such as the production of bacteriocins or other metabolites [21,22]. Moreover, the utilization of LAB in plant-based products as dairy analogs has been researched [23,24]. However, there are no studies on the protective potential of using microorganisms in RTE meat analogs.

Therefore, the focus of this study was evaluating the possibility of utilizing the different LAB in commercialized plant-based fermented dry-cured sausages. This strategy might improve the sausages’ safety and properties to imitate a meat product. For this purpose, challenge tests were carried out to evaluate the effect of LAB isolated from ripened products on *L. monocytogenes*, and sensory techniques were applied to analyze the impact of the most promising microorganism against *L. monocytogenes* on the organoleptic characteristics and acceptability of the final product.

## 2. Materials and Methods

### 2.1. Microbial Cultures

The commercial *Latilactobacillus curvatus* strain (Amerex, Madrid, Spain) used by the company that markets the meat analog used in this study was used to form a starter culture together with catalase-positive Gram-positive cocci (*Staphylococcus carnosus*). The rest of the LAB used are part of the collection of the Food Hygiene and Safety research group of the University of Extremadura (Table 1). These strains were isolated from “Torta del Casar” cheese and “salchichón”, a dry-fermented sausage, with proven activity against *L. monocytogenes* in cheese and “salchichón” model systems [25]. *L. monocytogenes* S7-2 (serotype 4b) was utilized from the collection of the National Institute of Agricultural and Food Research and Technology (INIA) Culture Collection (Madrid, Spain).

The cultures were retrieved from frozen stock (−80 °C) maintained in the same media, which included 20% (*w*/*v*) glycerol (Thermo Fisher Scientific, Waltham, MA, USA). To prepare the inoculum of *L. monocytogenes* and LAB, cultures were incubated in Brain Heart Infusion broth (BHI; Conda Pronadisa, Madrid, Spain) at 37 °C for 48 h and Man, Rogosa, and Sharpe broth (MRS; Conda Pronadisa, Madrid, Spain) at 30 °C for 48 h, respectively. After the incubation time, the culture broth was centrifuged in 50 mL polypropylene tubes (Scharlau Chemie S.A., Barcelona, Spain) at 10,621× *g* for 5 min, discarding the supernatant and suspending the precipitate in Phosphate Buffered Saline (PBS; 0.32 g sodium dihydrogen phosphate (Scharlau Chemie S.A., Barcelona, Spain), 1.09 g disodium hydrogen phosphate (Scharlau Chemie S.A., Barcelona, Spain) and 9 g NaCl (Labbox Labware S.L., Barcelona, Spain) per liter of distilled water) to be kept at 4 °C until use. The initial cell counts of *L. monocytogenes* and LAB were checked on the initial day of all assays on Ottaviani and Agosti agar plate culture (ALOA; Conda Pronadisa, Madrid, Spain) and MRS agar, respectively, of each of the evaluated experimental groups.

### 2.2. Preparation of the Plant-Based Analog and Experimental Design

A commercial RTE plant-based dry-cured fermented sausage was employed in the present study, being elaborated within the University of Extremadura facilities. The recipe provided by the industry that commercializes the meat analog evaluated in the present study was replicated, utilizing the following ingredients: potato, olive oil, shea butter, pea protein, dried red pepper, garlic, paprika, salt, vegetable fibers, and vegetal casing (United Caro, Granada, Spain). This company utilizes a starter culture in the analog production, but it was used in only one of the batches to faithfully reflect the actual characteristics of the product. The physicochemical and sensory characterization of the product has been previously performed [26]. 

**Figure 1:**
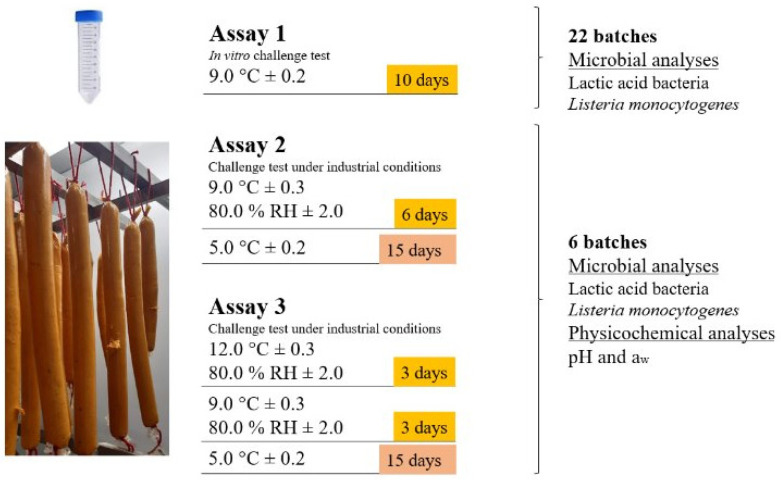
Scheme of the experimental design performed in the present study. The days highlighted with yellow represent the aerobic fermentation time, meanwhile, the days marked with red represent the vacuum-packed storage period.

Three consecutive assays, with a different analog elaboration for each, were carried out to facilitate their operation, summarized in Figure 1. Three independent sausages of each batch were analyzed in every assay (*n* = 3). The first one consisted of in vitro fermentation of the analog in 50 mL polypropylene tubes, allowing us to independently analyze the protective potential of 20 LAB and the starter culture used by the manufacturer against *L. monocytogenes*, and a control batch only inoculated with the pathogen. In this preliminary challenge test, the conditions were set at 9 °C ± 0.2 for 10 days. In the second assay, the analog was stuffed into the vegetal casings used by the plant-based analog manufacturer, and the fermentation took place in the pilot plant of the Faculty of Veterinary of the University of Extremadura for 6 days (9.0 °C ± 0.3 and 80.0% HR ± 2.0). The 5 LAB that gave the best results against *L. monocytogenes* in the in vitro assay were evaluated. Finally, the third assay was a two-step fermentation of 3 days (12.0 °C ± 0.3 and 80.0% HR ± 2.0), and 3 days (9.0 °C ± 0.3 and 80.0% HR ± 2.0) to test the impact of higher temperatures in the anti-*L. monocytogenes* activity of LAB on this novel plant-based product. Further, the fermented dry-cured sausages from assays 2 and 3 were vacuum-packed (Lavezzini S.R.L., Piacenza, Italy) and stored for 15 days at 5 °C ± 0.2, following the same procedure applied to the product after commercialization and aiming to replicate the typical temperatures found in household refrigerators [27,28]. Pouches had 90 μm thickness with an oxygen transmission rate under 70 cc per square meter per day at 23 °C and 0% RH, and a water vapor transmission rate under 10 g per square meter per day at 38 °C and 90% RH.

### 2.3. Challenge Tests

#### 2.3.1. In Vitro Assay

The plant-based analog was prepared the day before inoculation and kept at 4 °C. Twenty-two batches were prepared using three 50 mL polypropylene tubes per batch that were filled with approximately 25 g each. Twenty different LAB were tested; one of the batches was used as an uninoculated control (without *L. monocytogenes* or LAB), and another one was only inoculated with *L. monocytogenes*. All batches were sampled immediately after the inoculation of LAB, on MRS and ALOA agar, allowing LAB and *L. monocytogenes* counts to be performed after incubation for 48 h at 30 °C under microaerophilic conditions and 24–48 h at 37 °C, respectively. Final concentrations of ≈8 log CFU/g and ≈7 log CFU/g were achieved for LAB and *L. monocytogenes* on the initial day of fermentation, respectively. The challenge test took place for 10 days at 9 °C ± 0.2, after which each batch was sampled again.

#### 2.3.2. Assays Under Industrial Conditions

The plant-based analog was prepared the day before, as indicated in the in vitro assay, divided into 6 batches, 5 of which were inoculated with different LAB strains (*Llb. curvatus* commercial starter, *Lacticaseibacillus casei* 31, *Lacticaseibacillus paracasei* 74, *Lcb. casei* 116, and *Latilactobacillus sakei* 205) ≈6 log CFU/g and another one only inoculated with *L. monocytogenes*. A lower concentration of LAB was inoculated than in the in vitro assay to evaluate the industrial concentrations usually employed in meat-based dry-cured sausages. Each batch had a *L. monocytogenes* uninoculated twin, for pH and a_w_ evaluation. The samples inoculated with *L. monocytogenes* contained ≈4–5 log CFU/g of this pathogen. The impact of LAB on *L. monocytogenes* was evaluated using two assays; the first was conducted at 9 °C, which corresponds to the manufacturer’s recommended conditions, and the second at 12 °C, a higher temperature that could compromise the safety of the final product during dry-cured sausage fermentation.

On the initial day, the sausages from both assays were sampled, and microbial incubation was performed in different media as indicated in the in vitro assay. The sausages were fermented in the pilot plant located at the Faculty of Veterinary in a maturation chamber until they reached on day 6 the weight loss indicated by the industry, in the case of the first assay. On the 6th day, the sausages from both elaborations were sampled in the same way as on the initial day. Subsequently, all batches were vacuum packed and kept for 15 days at 5 °C to evaluate the development of *L. monocytogenes* during the expected refrigerated commercialization period of the product, performing a microbiological analysis identical to that which had been carried out on the previous sampling days.

### 2.4. Physicochemical Analyses

The physicochemical analyses were conducted exclusively on samples from the pilot plant trials, specifically those within each batch that were not inoculated with the pathogenic microorganism. The analyses followed the guidelines of the Association of Official Analytical Chemists [29]. A pH meter (Hach Lange Spain S.L.U., Barcelona, Spain) calibrated with 3 standard solutions (Scharlau Chemie S.A., Barcelona, Spain) was used to measure pH (JP SELECTA S.A., Barcelona, Spain). The a_w_ values were calculated at 25 °C using a specialized instrument (Novasina AG, Lachen, Switzerland) and standardized salt solutions with known a_w_ values.

### 2.5. Sensory Evaluation

The objective of the sensory analysis performed was to evaluate the differences between the commercial product and the one inoculated with the LAB that obtained the best results in the challenge test under industrial maturing conditions, *Llb. sakei* 205. In this case, a LAB uninoculated batch was also compared to justify the use of this type of microorganism in meat analogs. The sausages were fermented in the drying chamber (Oscar Zarzosa S.A., Burgos, Spain) of the experimental kitchen of the Meat and Meat Products Research Institute (IProCar). The fermentation conditions employed were the ones that obtained the best *L. monocytogenes* control, 9.0 °C ± 0.3 and 80.0% HR ± 2.0, until they reached the weight loss indicated by the industry on day 6.

The first test to be performed was a triangular test, which enables the evaluation of the differences between two products [30,31,32,33]. As three samples were available, three triangular tests were carried out, providing water and unsalted bread to clean the palate between samples (Control vs. Commercial; Control vs. 205; Commercial vs. 205). To guarantee the randomness of the responses, two models were designed for each of the tests, with the different sample being a different one in each of the models. The triangular test was developed in the facilities of the University of Extremadura, with the participation of 30 panelists.

Secondly, a Check-All-That-Apply (CATA) test was carried out to determine the characteristics of each evaluated product and consumer liking with a 5-point hedonic scale [34,35]. The attributes used (Table 2) were selected by compiling the scientific literature on meat products and a preliminary test with a focus group consisting of 4 expert panelists in meat and meat products [36,37]. To guarantee the randomness of the responses, three models of the test were carried out, in which the samples and the list of attributes were randomly presented. For the CATA test, a panel of 102 participants from 18 to 64 years of age was formed at the University of Extremadura facilities (personal staff, students, and visitors), who were given water and unsalted bread to clean the palate between samples. Before their participation, the panelists were informed of the purpose of the research and signed their consent to participate in the study.

### 2.6. Data Analysis

The microbiological results were statistically analyzed by calculating the normality of the data sets with the Shapiro–Wilk test, and the homogeneity of variances of the data sets with Levene’s statistic. Depending on the results of the normality and homogeneity tests, Student T test and Tukey’s HSD (*p* > 0.05) or Kruskal–Wallis K, Mann–Whitney’s U, and Dunnett’s T3 (*p* ≤ 0.05) tests were applied. To perform these statistical tests, IBM SPSS Statistics v.27 (IBM Co., New York, NY, USA) was used.

Regarding the sensory data, the triangular test results were statistically analyzed using a 1/3 binomial test. Cochran’s Q test was applied to the CATA results to study significant differences between samples for each of the attributes. Thereafter, post hoc pairwise comparisons between samples were made using the McNemar (Bonferroni) multiple comparisons procedure, using the 5% level of significance (*p* < 0.05). Correspondence analysis (CA) was performed to obtain the relationship between samples and terms from the CATA. Moreover, a penalty analysis (PA) was performed on CATA data considering the terms mentioned by at least 20% of panelists. In addition, a Principal Coordinate Analysis (PCoA) was carried out on acceptability and CATA attributes. The CATA test results were analyzed with the use of the XLSTAT 2023.2.0 software (Addinsoft, Paris, France). Finally, Tukey’s HSD parametric test was performed on acceptability results using IBM SPSS Statistics v.27 software.

## 3. Results and Discussion

### 3.1. In Vitro Assay

LAB and *L. monocytogenes* microbial counts were performed on the initial and final days of ripening (Table 3). LAB displayed a significant increment in nearly all batches analyzed over both days, including the non-inoculated batch, ultimately reaching final concentrations around 8–9 log CFU/g, which other authors consider the maximum levels typically observed in dry-cured fermented sausages [38,39,40]. In Table 3, it can be observed that the LAB-inoculated batches exhibited more homogeneous growth on the final sampling day, as indicated by a lower standard deviation compared to their uninoculated homologs, thereby achieving greater product standardization [41].

**Figure 2:**
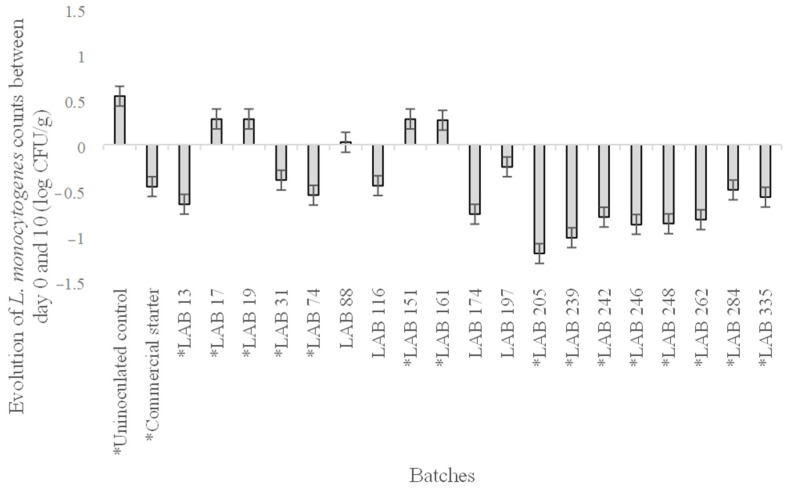
Evolution of the *Listeria monocytogenes* load (log CFU/g) for each batch between the initial (day 0) and final day (day 10) of fermentation. Statistical differences (*p* ≤ 0.05) between both sampling days within the same batch are expressed with an asterisk (*).

The *L. monocytogenes* counts demonstrated that the applied control strategy effectively slowed down the development of this pathogenic microorganism, as the control group without inoculated LAB underwent a growth of 0.55 log CFU/g, the highest among all sampled lots (Figure 2). Therefore, the inoculation of any LAB allowed the control of the development of *L. monocytogenes*, as has been demonstrated by several authors in other matured products [42,43,44]. This information also proved that the LAB isolated from a different environment than the inoculated batter in the present study can function efficiently. This has previously been proven by Cenci-Goga et al. [45], transferring milk-origin LAB to meat products. Even though batches inoculated with LAB 17, 19, 88, 151, and 161 obtained higher counts of *L. monocytogenes* at the end of the ripening period, the batch that obtained the best results was *L. sakei* 205, which generated a reduction of 1.2 log CFU/g since the initial fermentation time (Figure 2).

*Lcb. casei* 31, *Lcb. paracasei* 74, *Lcb. casei* 116, and *Llb. sakei* 205 were the other ones showing the highest *L. monocytogenes* reductions, around 0.5 log CFU/g (Figure 2). They were selected to perform the assays in the pilot plant based on the reductions achieved and the references to some of them in the scientific literature produced by the research group [46,47,48,49,50]. The commercial starter culture was also selected to compare its results.

### 3.2. Assay Under Industrial Conditions

The second challenge test was performed with ripening at 9 °C for 6 days and the third one at a higher temperature. LAB and *L. monocytogenes* microbial counts and physicochemical analyses were performed on the inoculation day, the final day of ripening, and after 15 days under commercialization conditions at 5 °C.

#### 3.2.1. Physicochemical Results

The a_w_ values were the same for both industrial assays despite the differences in the temperature of ripening, with initial values of 0.966, close to 0.960 on day 6, and around 0.956 on the final day of analysis (Table 4). However, pH presented differences between both challenge tests (*p* ≤ 0.05) for almost every measure, likely due to the differences in the action of the LAB at different fermentation temperatures. On the initial day, there were also significant differences, with 5.60 pH for the first assay, and 6.00 pH for the second one. On the 6th day, the pH of the uninoculated batch was the highest, around 5.60 and 6.10 for the first and second assays, respectively. On the other hand, the batch inoculated with LAB 205 obtained the lowest pH values, 4.64 and 4.87 for the first and second assays, respectively. It is remarkable how, on the final day of analysis, the batches inoculated with LAB 31, 116, and 205, did not obtain significant differences between both assays. Once again, the uninoculated batches resulted in the highest pH values on the last sampled day, 5.57 and 5.68 in the first and second assays, respectively. Moreover, the LAB-uninoculated batches showed different pH at the commercialization stage too, due to their lack of homogeneity [51]. The utilization of different starters showed different pH results, as has been indicated by other authors in meat products [52], with *L. sakei* 205 being the one that achieved lower pH levels in both assays.

Based on the physicochemical characteristics, the applicable microbiological criteria for *L. monocytogenes* established by the EU require the absence of this pathogenic bacterium in 25 g of product since, at the end of processing, the pH is above 4.4 and the combination of this parameter and the a_w_ value is never lower than 5.0 and 0.940, respectively [53]. The physicochemical characteristics of the product proved how, during the entire processing, the evaluated analog allowed the development of *L. monocytogenes* in either of the studied fermentation conditions. In this clean-label RTE product, in case of a lack of biocontrol measures, possible contamination with *L. monocytogenes* in the industry would pose a risk to the consumer [54]. This fact highlights the special attention to the establishment of effective control measures, such as the utilization of LAB evaluated in the present work.

#### 3.2.2. Microbial Results

The LAB reached different levels in both assays performed under industrial conditions, showing differences in almost every sampling day and across batches, as reflected in the pH results. The first assay reached levels of 9.0–9.6 log CFU/g and the second one among 7.5–9.0 log CFU/g at the end of the ripening time on day 6 (Table 5). In the LAB-inoculated batches of both assays, the LAB concentration was between 6 and 7 log CFU/g, though the uninoculated batches started with levels below 5.5 log CFU/g in both assays. A larger difference was found between the LAB-uninoculated and LAB-inoculated batches, once again proving a higher homogeneity in the latter. The lack of LAB inoculation did not pose a problem to the development of LAB in the LAB-uninoculated batch. In both challenge tests, it can be observed how this product, made only with vegetable materials, allowed the development of LAB isolated from animal products. In the first assay, a LAB concentration of 9–9.5 log CFU/g was reached after 6 days at 9 °C. In the second assay, the LAB levels reached 7.5–9 log CFU/g after 6 days at 12 °C. Statistical differences (*p* ≤ 0.05) were observed between both assays in every batch at day 6. On day 15 under vacuum, a significant reduction (*p* ≤ 0.05) in the concentration of LAB was observed concerning the end of ripening, day 6, in some of the batches of the first assay. This fact has been described in previous studies without definitive conclusions, as some authors have observed an increase in LAB concentration during vacuum chilling, while others have noted a decrease [40,55]. Thus, the LAB levels of both assays were found to be between 6.91 and 9.17 log CFU/g, maintaining only the higher counts of the first assay (*p* ≤ 0.05) in the LAB-uninoculated batches and that inoculated with LAB 74.

*L. monocytogenes* levels around 4–5 log CFU/g were inoculated in both assays. The *L. monocytogenes* concentration of the LAB-uninoculated batches increased by 1.43 log CFU/g during the initial days of the first assay, and 0.59 log CFU/g in the second one, probably due to the higher initial inoculation in this case. The *L. monocytogenes* counts proved a significant increase (*p* ≤ 0.05) over 1 log CFU/g in the concentration of all the batches analyzed during the challenge test in the first six days of ripening, except for the one inoculated with *Llb. sakei* 205 in the first assay and the inoculated with *Lcb. casei* 116 in the second one. This fact points out that LAB 205 works better at lower temperatures than LAB 116. However, *Llb. sakei* 205 achieved a significant reduction (*p* ≤ 0.05) of 1.2 logarithmic units during the 6 fermentation days at 9 °C (Table 6), while LAB 116 achieved a significant reduction (*p* ≤ 0.05) of 0.8 logarithmic units during the 6 fermentation days at 12 and 9 °C. This means that *Llb. sakei* 205 achieved the highest reduction in *L. monocytogenes* counts, exceeding two logarithmic units compared to the control batch (LAB-uninoculated batch) in the absence of preventive measures by the sixth day of processing. This protective culture has been evaluated and identified as a bacteriocin producer in in vitro assays [47]. The *L. monocytogenes* reduction achieved by the LAB demonstrates that the protective activity of *Llb. sakei* 205, as reported by Martín et al. [49] in a meat-based dry-cured fermented sausage, can be transferred to a plant-based analog. The production of bacteriocins by *Llb. sakei* 205 might justify the protective effect against *L. monocytogenes* in comparison with other strains, though further investigation must be performed to unveil the effectiveness of LAB 205.

On the commercialization sampling day, after 15 days under oxygen limitation conditions, *L. monocytogenes* reduction can be observed in all inoculated batches from both assays concerning the initial and the final day of ripening, except the LAB-uninoculated batch that increased 2.86 log CFU/g and 0.82 CFU/g in the first and second assays, respectively. Therefore, vacuum packaging generates a favorable environment for the tested LAB, enhancing their activity against *L. monocytogenes*, with *Llb. sakei* 205 once again producing the greatest reduction in both challenge tests. This fact proves once again that LAB 205 performs better under lower temperature conditions than *Lcb. casei* 116. In the first assay, a reduction of 1.97 log CFU/g was achieved concerning the initial day of ripening, while in the second one, only a reduction of 0.82 log CFU/g was achieved. Studies based on meat products have also shown how the presence of *L. monocytogenes* decreases over time in the absence of oxygen [40,56,57,58]. However, the tendency was not observed in the absence of the selected LAB in the evaluated plant-based analog, as can be noticed in Table 6.

The use of *Llb. sakei* 205 has also been studied by Martín et al. [49] in meat-based dry-cured fermented sausages, achieving a 2 log CFU/g reduction in *L. monocytogenes* counts after 90 days of maturation. In the present study, it is noteworthy that the inoculated LAB achieved a reduction in the pathogenic microorganism of 1.17 log CFU/g within 6 days, whereas Martín et al. [49] reported a reduction of only 0.34 log CFU/g over their shortest sampling period of 15 days.

The lack of scientific literature that investigates the utilization of biocontrol starters in RTE novel analogs establishes the importance of the present study, which not only tries different LAB to find the one that better suits the analyzed product but also compares its action against the lack of an inoculated LAB. The use of *Llb. sakei* 205 during the first assay under industrial conditions established an *L. monocytogenes* reduction of 2.60 log CFU/g against the uninoculated batch on the 6th day of ripening, which lacks biocontrol measures in the studied clean label RTE plant-based analog. In the case of the second assay under industrial conditions, the reduction achieved by LAB 116 was 1.35 log CFU/g against the uninoculated batch.

The differences in *L. monocytogenes* biocontrol were increased in the case of the commercialization stages, in which the use of *Llb. sakei* 205 achieved a reduction of 3.83 log CFU/g against the uninoculated batch in the first assay under industrial conditions. In the second assay, larger reductions were also achieved by LAB 205 with a *L. monocytogenes* count decrease of 1.62 log CFU/g.

The levels of contamination typically found in dry-cured fermented sausages within the industry are reported in the literature to reach a maximum of 1–2 log CFU/g [57,59,60]. Thus, the use of *L. sakei* 205 offers a clean label solution to address the common contaminations that may occur during processing, representing a strategy aligned with current consumer demands for reduced use of food additives [14]. However, the utilization of LAB cannot be the only strategy that food facilities employ against *L. monocytogenes* contamination. Other strategies, such as the implementation of an environmental monitoring program, must be carried out to ensure microbial safety for the end consumer [61]. The use of this LAB could be proposed as a preventive measure in the context of the HACCP plan. The *L. monocytogenes* reduction in the first pilot plant assay justified selecting these ripening conditions and LAB 205 inoculation for sensory evaluation, as the use of different microorganisms might improve product sensory characteristics [62].

### 3.3. Sensory Evaluation

In the sensory evaluation carried out, an uninoculated batch was compared with the one inoculated with the commercial culture, as well as with *Llb. sakei* 205, which was the bacterium that obtained the most effective microbiological control of *L. monocytogenes* under all ripening conditions at refrigeration temperatures. All batches used in the sensory evaluation were microbiologically sampled for the absence of *L. monocytogenes* in 25 g of product [63].

The discriminative sensory test (triangular) presented differences (*p* ≤ 0.05) between the LAB-uninoculated batch and the LAB-inoculated batches individually. Seventy and eighty percent of the panelists correctly identified the odd sample in the triangle tests when the uninoculated batch was presented alongside either the commercial batch or *Llb. sakei* 205, respectively. On the other hand, in the discriminative tests in which the samples inoculated with any LAB were confronted, only 43% of the panelists identified the odd sample (*p* > 0.05). Table 7 shows differences in the citation frequencies of the pairwise multiple comparisons, which may describe the differences found during the discriminatory test. The uninoculated batch was classified as having less acid taste than the rest, and with a less intense saltiness, and cured flavor, but a higher rancid flavor than the batch inoculated with the *Llb. sakei* 205, probably due to the lipid oxidation intensified by LAB [64]. Although it was expected to have a greater meaty flavor in the batches in which starter cultures are used due to greater enzymatic hydrolysis [62,65] or a sweeter flavor in the batch in which the commercial culture is used due to the inoculation of Gram-positive catalase-positive cocci [66], there were no differences in the citation of these attributes between batches. Though a future volatile compound description should be performed, lactic acid bacteria might reduce the concentration of aldehydes resulting from lipid oxidation [67]. The greater perception of acid taste is related to the use of starter cultures [41], as can also be seen in the physicochemical results. Regarding the remaining attributes for which significant differences were found, no literature has been found to justify their greater intensity in the batch with *Llb. sakei* 205 compared to the uninoculated control. However, these attributes could be correlated with acidic perception or might result directly from differences in the metabolism of the inoculated LAB [62,65,66]. Despite the small sensory characteristics that differentiate the samples, there was no influence on their acceptability. The acceptability results presented the highest averages for the inoculated products, but there are no statistical differences (*p* ≤ 0.05) between them (Table 7).

**Figure 3:**
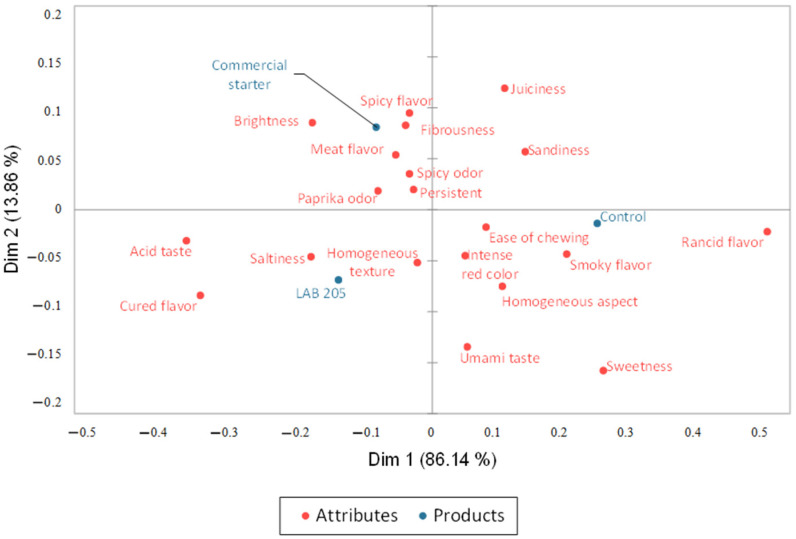
Correspondence analysis of the Check All That Apply term frequencies for the evaluated samples.

The Correspondence Analysis (CA) displays the attributes that define each of the samples analyzed (Figure 3). Dimension 1 explained 86.14% of the total variance, while dimension 2 explained 13.86%. The control batch stands out for the greater influence of the rancid flavor attribute, which differs significantly (*p* ≤ 0.05) from the batch inoculated with *Llb. sakei* 205. This batch is found in the same quadrant as ease of chewing, smoky flavor, saltiness, homogeneous texture, cured flavor, and acid taste, showing some statistical differences in their citation frequencies (Table 7). The batch inoculated with the commercial starter is found closer to spicy flavor, fibrousness, brightness, and meat flavor, though none of them showed statistical differences with any of the other analyzed samples.

**Figure 4:**
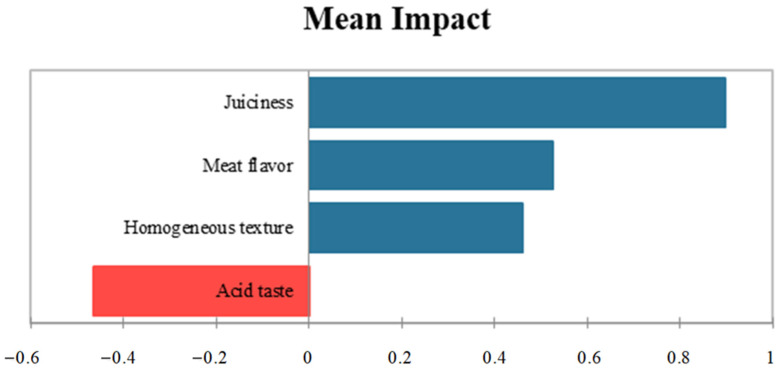
Penalty analysis in which the drivers of liking of the samples analyzed during the Check All That Apply test are shown.

Figure 4 displays the drivers of acceptability in a penalty analysis (PA). Acid taste has been identified as a factor contributing to disliking, decreasing overall acceptability by 0.46 points out of 5. In contrast, meat flavor, juiciness, and homogeneous texture are positively associated with overall liking, increasing acceptability by 0.52, 0.89, and 0.45 points out of 5, respectively. Although the uninoculated sample exhibited a significantly lower acid taste (*p* ≤ 0.05) compared to the other batches, the one inoculated with *Llb. sakei* 205 was found with a closer relation in the CA. This suggests that the adverse effect of reduced acidity may disproportionately influence its sensory profile. On the other hand, meat flavor was located in the same quadrant as the sample inoculated with the commercial starter, suggesting that the higher frequency of this attribute’s identification in the batch inoculated with the commercial starter may have had a positive impact.

The PA established some key points for improving the acceptability of the meat analog, such as increasing juiciness, meat flavor, and homogeneous texture, while at the same time decreasing acidity in the final product. In studies on meat products, juiciness is also considered an essential attribute for increasing the acceptability of the product, while acidity is a driver of dislike [36,68]. In the case of the evaluated analog, these parameters could be modified by controlling the moisture loss of the product during ripening and the inoculation of different microorganisms, though any of the currently employed LAB controlled the acidity production enough [62].

**Figure 5:**
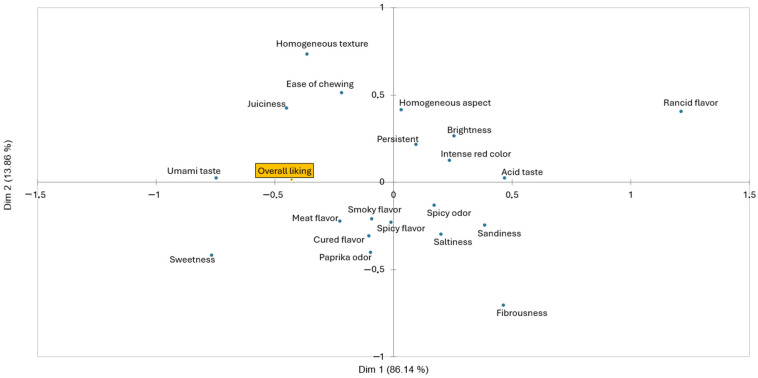
Principal coordinate analysis plot of Check All That Apply term frequencies.

PCoA showed that umami taste, meat flavor, smoky flavor, persistence, and juiciness are the attributes that define the overall liking of the meat analog evaluated in the present study (Figure 5). Though the hedonic test showed no significant differences between the analyzed samples, PCoA may help to identify the samples closer to a higher overall liking. However, pairwise multiple comparisons did not display significant differences in any of these attributes. Moreover, CA did not show any relevant additional information, as the meat flavor was closer to the sample with the commercial starter, but the smoky flavor more closely resembled that of the control uninoculated sample. As no batch showed significantly higher overall liking, LAB 205 is recommended for the plant-based analog to enhance microbial control in this clean-label RTE product.

Further steps include the combined utilization of *Lcb. casei* 116 and *Llb. sakei* 205 to achieve protection against *L. monocytogenes* at high and low fermentation temperatures, with its corresponding sensory evaluation to assess its organoleptic impact. Moreover, based on the sensory results obtained in the present study, the formulation of the evaluated product might be improved to increase the acceptability of the plant-based analog.

## 4. Conclusions

The use of protective cultures is an effective, cheap, and easy-to-apply technology widely used in other ripened products. The utilization of lactic acid bacteria as a clean-label biocontrol method against *Listeria monocytogenes* has been proven to be a successful tool in the plant-based dry-cured fermented sausages evaluated. Specifically, *Latilactobacillus sakei* 205 achieved an *L. monocytogenes* reduction of 2.6 log CFU/g by the end of ripening compared to the uninoculated batch, and no differences were found in the overall liking of the product inoculated with this microorganism relative to the currently commercialized product. Therefore, it is recommended to use *Llb. sakei* 205 as a protective culture in the plant-based product of study at the conditions studied in the first industrial assay. The upcoming steps should involve the concurrent use of *Lacticaseibacillus casei* 116 and *Llb. sakei* 205 to establish protection against *L. monocytogenes*, addressing both high and low fermentation temperatures, as a precautionary measure in the event of an inadvertent temperature increase.

## Figures and Tables

**Table 1:** Lactic acid bacteria (LAB) used in the present study.

Codification	LAB Species
Commercial starter	*Latilactobacillus curvatus*
13, 74, 239, 242, 248, 262, 335	*Lacticaseibacillus paracasei*
17, 31, 88, 116, 246	*Lacticaseibacillus casei*
19, 174, 197, 205	*Latilactobacillus sakei*
151	*Lactococcus garvieae*
161	*Enterococcus durans*
284	*Lactiplantibacillus plantarum*

**Table 2:** List of attributes employed in the Check-All-That Apply test.

	Attributes	
Acid taste	Smoky flavor	Juiciness
Sandiness	Meat flavor	Paprika odor
Brightness	Cured flavor	Spicy odor
Sweet taste	Rancid flavor	Persistent
Ease of chewing	Homogeneous aspect	Saltiness
Fibrousness	Homogeneous texture	Umami taste
Spicy flavor	Intense red color	

**Table 3:** LAB counts from the in vitro assay, fermented at 9 °C for 10 days, on the initial day and final day of fermentation.

Batches	Initial Day (log CFU/g)	Final Day (log CFU/g)
Uninoculated control	4.98 ± 0.02 *	7.89 ± 0.82 *
*Listeria monocytogenes*	4.98 ± 0.02 *	7.89 ± 0.82 *
*L. monocytogenes* and commercial starter	8.78 ± 0.03 *	9.02 ± 0.09 *
*L. monocytogenes* and LAB 13	8.75 ± 0.06	8.85 ± 0.21
*L. monocytogenes* and LAB 17	8.51 ± 0.01 *	9.06 ± 0.06 *
*L. monocytogenes* and LAB 19	8.53 ± 0.02 *	8.98 ± 0.07 *
*L. monocytogenes* and LAB 31	8.37 ± 0.04 *	8.49 ± 0.06 *
*L. monocytogenes* and LAB 74	8.82 ± 0.10	8.75 ± 0.10
*L. monocytogenes* and LAB 88	8.18 ± 0.10 *	8.57 ± 0.19 *
*L. monocytogenes* and LAB 116	9.23 ± 0.12 *	8.76 ± 0.10 *
*L. monocytogenes* and LAB 151	8.55 ± 0.01	8.40 ± 0.38
*L. monocytogenes* and LAB 161	9.05 ± 0.05 *	8.59 ± 0.07 *
*L. monocytogenes* and LAB 174	6.78 ± 0.08 *	8.70 ± 0.12 *
*L. monocytogenes* and LAB 197	7.33 ± 0.09 *	8.69 ± 0.10 *
*L. monocytogenes* and LAB 205	6.92 ± 0.07 *	8.61 ± 0.13 *
*L. monocytogenes* and LAB 239	9.09 ± 0.02 *	8.33 ± 0.04 *
*L. monocytogenes* and LAB 242	9.03 ± 0.01 *	8.27 ± 0.14 *
*L. monocytogenes* and LAB 246	9.25 ± 0.03 *	8.15 ± 0.15 *
*L. monocytogenes* and LAB 248	9.36 ± 0.07 *	8.31 ± 0.14 *
*L. monocytogenes* and LAB 262	9.24 ± 0.04 *	8.43 ± 0.23 *
*L. monocytogenes* and LAB 284	8.85 ± 0.02 *	8.61 ± 0.09 *
*L. monocytogenes* and LAB 335	9.07 ± 0.03 *	8.61 ± 0.14 *

Results are expressed as mean ± standard deviation. Statistical differences (*p* ≤ 0.05) between both sampling days within the same batch, at the same sampling day, are expressed with an asterisk in the same row (*).

**Table 4:** Physicochemical results for each evaluated batch at the initial (day 0) and final day (day 6) of ripening, and after 15 days under commercialization conditions after ripening (vacuum packed and stored at 5 °C).

**First assay under industrial conditions (9 °C)**			
**Batches**	**Day 0**	**Day 6**	**Day 6 + 15**
**pH**			
**Uninoculated batch**	5.60 ± 0.01 *	5.64 ± 0.12 ^4^*	5.57 ± 0.03 ^5^*
**Commercial starter**	5.60 ± 0.01 ^b^*	4.97 ± 0.04 ^a2^	4.97 ± 0.06 ^a4^*
**LAB 31**	5.60 ± 0.01 ^c^*	5.31 ± 0.04 ^b3^*	4.34 ± 0.07 ^a1^
**LAB 74**	5.60 ± 0.01 ^c^*	4.90 ± 0.01 ^b2^*	4.12 ± 0.08 ^a12^*
**LAB 116**	5.60 ± 0.01 ^c^*	4.90 ± 0.11 ^b2^*	4.55 ± 0.14 ^a23^
**LAB 205**	5.60 ± 0.01 ^b^*	4.64 ± 0.03 ^a1^*	4.72 ± 0.09 ^a34^
**a_w_**			
**Uninoculated batch**	0.966 ± 0.001 ^b^	0.960 ± 0.001 ^ab^	0.957 ± 0.001 ^a^
**Commercial starter**	0.966 ± 0.001 ^b^	0.959 ± 0.001 ^ab^	0.954 ± 0.002 ^a^
**LAB 31**	0.966 ± 0.001 ^b^	0.959 ± 0.001 ^ab^	0.954 ± 0.002 ^a^
**LAB 74**	0.966 ± 0.001 ^b^	0.958 ± 0.001 ^ab^	0.956 ± 0.001 ^a^
**LAB 116**	0.966 ± 0.001 ^b^	0.958 ± 0.001 ^ab^	0.956 ± 0.002 ^a^
**LAB 205**	0.966 ± 0.001 ^b^	0.958 ± 0.001 ^ab^	0.954 ± 0.002 ^a^
**Second assay under industrial conditions (12 + 9 °C)**			
**Batches**	**Day 0**	**Day 6**	**Day 6 + 15**
**pH**			
**Uninoculated batch**	6.00 ± 0.01 *	6.11 ± 0.12 ^3^*	5.68 ± 0.03 ^3^*
**Commercial starter**	6.00 ± 0.01 ^c^*	4.93 ± 0.04 ^b12^	4.69 ± 0.06 ^a23^*
**LAB 31**	6.00 ± 0.01 ^c^*	5.11 ±0.04 ^b123^*	4.47 ± 0.07 ^a1^
**LAB 74**	6.00 ± 0.01 ^b^*	5.26 ± 0.01 ^ab23^*	4.47 ± 0.08 ^a12^*
**LAB 116**	6.00 ± 0.01 ^c^*	5.19 ± 0.11 ^b23^*	4.56 ± 0.14 ^a12^
**LAB 205**	6.00 ± 0.01 ^b^*	4.87 ± 0.03 ^a1^*	4.60 ± 0.09 ^a123^
**a_w_**			
**Uninoculated batch**	0.966 ± 0.001 ^b^	0.960 ± 0.001 ^ab^	0.957 ± 0.001 ^a^
**Commercial starter**	0.966 ± 0.001 ^b^	0.959 ± 0.001 ^ab^	0.954 ± 0.002 ^a^
**LAB 31**	0.966 ± 0.001 ^b^	0.959 ± 0.001 ^ab^	0.954 ± 0.002 ^a^
**LAB 74**	0.966 ± 0.001 ^b^	0.958 ± 0.001 ^ab^	0.956 ± 0.001 ^a^
**LAB 116**	0.966 ± 0.001 ^b^	0.958 ± 0.001 ^ab^	0.956 ± 0.002 ^a^
**LAB 205**	0.966 ± 0.001 ^b^	0.958 ± 0.001 ^ab^	0.954 ± 0.002 ^a^

Results are expressed as mean ± standard deviation. Statistical differences (*p* ≤ 0.05) among the sampling days within the same batch are expressed with a different letter as superscripts in the same row (^a–c^). Statistical differences (*p* ≤ 0.05) among the batches within the same assay, on the same sampling day, are expressed with a different number as superscripts in the same row (^1–5^). Statistical differences (*p* ≤ 0.05) between both assays within the same batch and sampling day are expressed with an asterisk as superscripts (*).

**Table 5:** Lactic acid bacteria evolution for each evaluated batch at the initial (day 0) and final day (day 6) of ripening, and after 15 days under commercialization conditions after ripening (vacuum packaged and 5 °C storage).

Batches	Day 0 (log CFU/g)	Day 6 (log CFU/g)	Day 6 + 15 (log CFU/g)
First assay under industrial conditions (9 °C)			
Uninoculated control	4.87 ± 0.03 ^a1^*	9.59 ± 0.12 ^c2^*	8.78 ± 0.04 ^b12^*
*L. monocytogenes*	5.52 ± 0.03 ^a2^*	9.33 ± 0.29 ^c12^*	8.54 ± 0.09 ^b1^*
*L. monocytogenes* and commercial starter	6.59 ± 0.26 ^a3^	9.28 ± 0.10 ^b12^*	8.88 ± 0.15 ^b12^
*L. monocytogenes* and LAB 31	6.61 ± 0.03 ^a3^*	9.44 ± 0.18 ^c12^*	8.99 ± 0.15 ^b12^
*L. monocytogenes* and LAB 74	6.44 ± 0.07 ^a3^*	9.07 ± 0.19 ^b1^*	9.17 ± 0.15 ^b2^*
*L. monocytogenes* and LAB 116	6.49 ± 0.08 ^a3^*	9.31 ± 0.08 ^b12^*	9.10 ± 0.31 ^b2^
*L. monocytogenes* and LAB 205	6.43 ± 0.09 ^a3^*	9.02 ± 0.04 ^b1^*	8.71 ± 0.29 ^b12^
Second assay under industrial conditions (12 + 9 °C)			
Uninoculated control	5.44 ± 0.03 ^a1^*	7.53 ± 0.03 ^b1^*	7.54 ± 0.24 ^b12^*
*L. monocytogenes*	5.44 ± 0.03 ^a1^*	7.87 ± 0.14 ^b1^*	6.91 ± 0.72 ^ab1^*
*L. monocytogenes* and commercial starter	6.44 ± 0.26 ^a123^	9.06 ± 0.06 ^b3^*	9.07 ± 0.05 ^b3^
*L. monocytogenes* and LAB 31	6.80 ± 0.03 ^a3^*	8.62 ± 0.09 ^b2^*	8.78 ± 0.09 ^b23^
*L. monocytogenes* and LAB 74	6.86 ± 0.07 ^a3^*	8.56 ± 0.22 ^b123^*	8.87 ± 0.04 ^b23^*
*L. monocytogenes* and LAB 116	7.14 ± 0.08 ^a3^*	8.51 ± 0.09 ^b2^*	8.82 ± 0.11 ^c23^
*L. monocytogenes* and LAB 205	6.05 ± 0.09 ^a2^*	8.87 ± 0.04 ^b23^*	8.82 ± 0.12 ^b23^

Results are expressed as mean ± standard deviation. Statistical differences (*p* ≤ 0.05) among the sampling days within the same batch are expressed with a different letter as superscripts in the same row (^a–c^). Statistical differences (*p* ≤ 0.05) among the batches within the same assay, on the same sampling day, are expressed with a different number as superscripts in the same row (^1–3^). Statistical differences (*p* ≤ 0.05) between both assays within the same batch and sampling day are expressed with an asterisk as superscripts (*).

**Table 6:** *Listeria monocytogenes* evolution for each evaluated batch at the initial (day 0) and final day (day 6) of ripening, and after 15 days under commercialization conditions after ripening.

Batches	Day 0 (log CFU/g)	Day 6 (log CFU/g)	Day 6 + 15 (log CFU/g)
First assay under industrial conditions (9 °C)			
*L. monocytogenes*	3.74 ± 0.06 ^a1^*	5.17 ± 0.10 ^b2^*	5.60 ± 0.16 ^b3^*
*L. monocytogenes* and commercial starter	4.15 ± 0.02 ^b2^*	5.46 ± 0.13 ^c2^*	2.59 ± 0.26 ^a1^*
*L. monocytogenes* and LAB 31	4.36 ± 0.03 ^b3^*	5.34 ± 0.33 ^c2^*	3.58 ± 0.56 ^a123^*
*L. monocytogenes* and LAB 74	4.53 ± 0.04 ^b4^*	5.59 ± 0.21 ^c2^*	3.02 ± 0.22 ^a1^*
*L. monocytogenes* and LAB 116	4.31 ± 0.09 ^b23^*	5.47 ± 0.23 ^c2^*	4.00 ± 0.15 ^a2^*
*L. monocytogenes* and LAB 205	4.15 ± 0.10 ^b2^*	2.98 ± 0.05 ^a1^*	2.18 ± 0.30 ^a1^*
Second assay under industrial conditions (12 + 9 °C)			
*L. monocytogenes*	5.33 ± 0.06 ^a^*	5.92 ± 0.33 ^ab2^*	6.15 ± 0.02 ^b2^*
*L. monocytogenes* and commercial starter	5.35 ± 0.02 ^a^*	6.21 ± 0.06 ^b2^*	4.62 ± 0.24 ^a1^*
*L. monocytogenes* and LAB 31	5.22 ± 0.03 ^a^*	6.39 ± 0.03 ^b2^*	5.09 ± 0.11 ^a1^*
*L. monocytogenes* and LAB 74	5.32 ± 0.04 ^b^*	6.34 ± 0.09 ^c2^*	4.90 ± 0.01 ^a1^*
*L. monocytogenes* and LAB 116	5.32 ± 0.09 ^b^*	4.55 ± 0.17 ^a1^*	4.54 ± 0.26 ^a1^*
*L. monocytogenes* and LAB 205	5.26 ± 0.10 ^b^*	6.16 ± 0.12 ^c2^*	4.44 ± 0.18 ^a1^*

Results are expressed as mean ± standard deviation. Statistical differences (*p* ≤ 0.05) among the sampling days within the same batch are expressed with a different letter as superscripts in the same row (^a–c^). Statistical differences (*p* ≤ 0.05) among the batches within the same assay, on the same sampling day, are expressed with a different number as superscripts in the same row (^1–4^). Statistical differences (*p* ≤ 0.05) between both assays within the same batch and sampling day are expressed with an asterisk as superscripts (*).

**Table 7:** McNemar (Bonferroni) pairwise multiple comparisons frequency of citation and acceptability over a 5-point scale of each sample analyzed.

Attributes	Control	Commercial Starter	LAB 205
Acid taste	0.167 ^a^	0.412 ^b^	0.480 ^b^
Brightness	0.088	0.157	0.137
Cured flavor	0.078 ^a^	0.167 ^ab^	0.216 ^b^
Ease of chewing	0.833	0.775	0.784
Fibrousness	0.088	0.118	0.098
Homogeneous aspect	0.363	0.294	0.343
Homogeneous texture	0.343	0.363	0.412
Intense red color	0.324	0.304	0.333
Juiciness	0.373	0.402	0.284
Meat flavor	0.216	0.284	0.255
Paprika odor	0.373	0.490	0.480
Persistent	0.206	0.245	0.235
Rancid flavor	0.265 ^b^	0.127 ^ab^	0.108 ^a^
Saltiness	0.245 ^a^	0.363 ^ab^	0.422 ^b^
Sandiness	0.343	0.324	0.265
Smoky flavor	0.324	0.235	0.245
Spicy flavor	0.343	0.461	0.373
Spicy odor	0.333	0.412	0.382
Sweet taste	0.127	0.069	0.098
Umami taste	0.098	0.078	0.108
Acceptability	2.76 ± 1.17	3.04 ± 1.07	3.06 ± 1.15

Cochran’s Q test was applied to the CATA results to study significant differences between samples for each of the attributes. Acceptability results are expressed as mean ± standard deviation. Statistical differences (*p* ≤ 0.05) among batches within the same attribute are expressed with a letter as superscripts in the same row (^a–b^).

## Data Availability

The original contributions presented in this study are included in the article. Further inquiries can be directed to the corresponding author(s).
